# A Discrepancy in the Transactions

**Published:** 1891-09

**Authors:** 


A Discrepancy Explained.—In the Transactions Texas State Medical Association, 1891, just issued, there is a discrepancy between the Report of Publishing Committee and the Treasurer's account, on page 39, in connection with the Transactions of 1890. It is caused by the Treasurer including two bills in one, under head of "paid for printing Transactions 1890."

The gross cost of printing the Transactions as per bill in hands of Treasurer, was $554.20. This included printing and binding, extra work on rule and figure work, lost time, changes after matter was set up, President's picture, manilla wrappers printed, twine, labor in wrapping, etc.

The other bill was for 6000 addresses to the profession, and 6000 petitions and circular letters, printed by order of the Committee on Medical Legislation, $115, and $6 for letter heads for Secretary's office. If this amount, $121, be deducted from $667, as entered by Treasurer, and cost of exchange added, it will be seen that the Transactions "cost about 84c. per volume of 344 pages," as stated in report of Publishing Committee, and not over a dollar each, as would appear from the Treasurer's report.

